# Successful Management of Teeth with Different Types of Endodontic-Periodontal Lesions

**DOI:** 10.1155/2018/7084245

**Published:** 2018-05-29

**Authors:** Hind Alquthami, Abdulaziz M. Almalik, Faisal F. Alzahrani, Lana Badawi

**Affiliations:** ^1^Department of Dentistry, Division of Endodontics, Prince Sultan Military Medical City, P.O. Box 7897, Riyadh 11159, Saudi Arabia; ^2^Department of Dentistry, Division of Periodontics, Prince Sultan Military Medical City, Riyadh, Saudi Arabia; ^3^Department of Dentistry, Division of Orthodontics, Prince Sultan Military Medical City, Riyadh, Saudi Arabia; ^4^Department of Dentistry, Division of Endodontics, Prince Sultan Military Medical City, Riyadh, Saudi Arabia

## Abstract

Endodontic-periodontal diseases often present great challenges to the clinician in their diagnosis, management, and prognosis. Understanding the disease process through cause-and-effect relationships between the pulp and supporting periodontal tissues with the aid of rational classifications leads to successful treatment outcomes. In this report, we present several treatment modalities in patients with different endodontic-periodontal lesions. A modification to the new endodontic-periodontic classification, Al-Fouzan's classification, was also added. The first case was classified as retrograde periodontal disease (i.e., primary endodontic lesion with drainage through the periodontal ligament). The second case was diagnosed as an iatrogenic periodontal lesion caused by root perforation. The third case was diagnosed as an iatrogenic periodontal lesion caused by tooth trauma due to orthodontic treatment. The first two cases were managed with a nonsurgical approach, whereas the third case was managed with nonsurgical and surgical approaches. All patients showed complete healing of soft and hard tissue lesions. A thorough understanding of the disease history and the patient's signs and symptoms, complete examination with full investigation, and the use of a systematic step-by-step approach in the management of such challenging endodontic-periodontal lesions with regular recall visits were very useful and successful.

## 1. Introduction

An endodontic-periodontal lesion consists of concurrent pulpal and periodontal disease in the same tooth. The spread of infection between the dental pulp and the periodontal ligament can occur through the apical foramen, lateral canals, dentinal tubules, and palatogingival grooves. Moreover, nonanatomic factors can have a role in this communication such as iatrogenic root canal perforations or a vertical root fracture. These pathways cause the spread of infection and bone destruction in a coronal-to-apical direction in the case of periodontal infection or in an apical-to-coronal direction in the case of endodontic infection [1].

In 1964, Simring and Goldberg [2] were the first to describe the relationship between pulpal and periodontal disease and referred to it as an “endo-perio lesion.” The most common classifications of endodontic-periodontal lesions were primary endodontic disease, primary periodontal disease, and combined disease, depending on the cause of the lesion [3]. In 2014, Al-Fouzan [4] suggested a new endodontic-periodontal interrelationship classification, based on the primary disease and its secondary effect. The classification is as follows.

1. Retrograde periodontal disease
  - Primary endodontic lesion with drainage through the periodontal ligament
  - Primary endodontic lesion with secondary periodontal involvement
2. Primary periodontal lesion
3. Primary periodontal lesion with secondary endodontic involvement
4. Combined endodontic-periodontal lesion
5. Iatrogenic periodontal lesion

This rational classification increases the understanding of the disease process and its origin. This understanding is essential in determining the correct diagnosis and providing treatment with predictable success. The success rate of joined endodontic-periodontal lesions without a regenerative procedure is between 27% and 37% [5]. These rates are much lower than the success rate of 95% with conventional nonsurgical root canal therapy [6]. The use of barrier membranes and/or bone-grafting materials during treatment encourages the growth of surrounding lost tissues such as the periodontal ligament, bone cementum, and connective tissue while preventing unwanted cell types such as epithelial cells [7]. The aim of this paper was to present the diagnosis and management of different endodontic-periodontal disease conditions with or without the use of regenerative bone techniques.

## 2. Case Presentations

### 2.1. Case 1: Retrograde Periodontal Disease: A Primary Endodontic Lesion with Drainage through the Periodontal Ligament

A 55-year-old woman with a noncontributory medical history was referred to the endodontic specialist clinic at Prince Sultan Military Medical City (PSMMC; Riyadh, Saudi Arabia) complaining of intraoral sinus with pus drainage in the right mandibular molar area. Clinical and radiographic examinations revealed a large amalgam restoration with recurrent caries and periapical and furcal radiolucency related to tooth #46. There was localized swelling in the gingival sulcus and the sinus in the gingival area. Tooth mobility was grade II. A midbuccal area with a narrow periodontal pocket > 10 mm was noted. The tooth had a negative response to the thermal vitality test (Endo-Ice; Hygenic Corp., Akron, OH, USA). The diagnosis was a necrotic pulp and chronic apical abscess.

**Figure 1:**
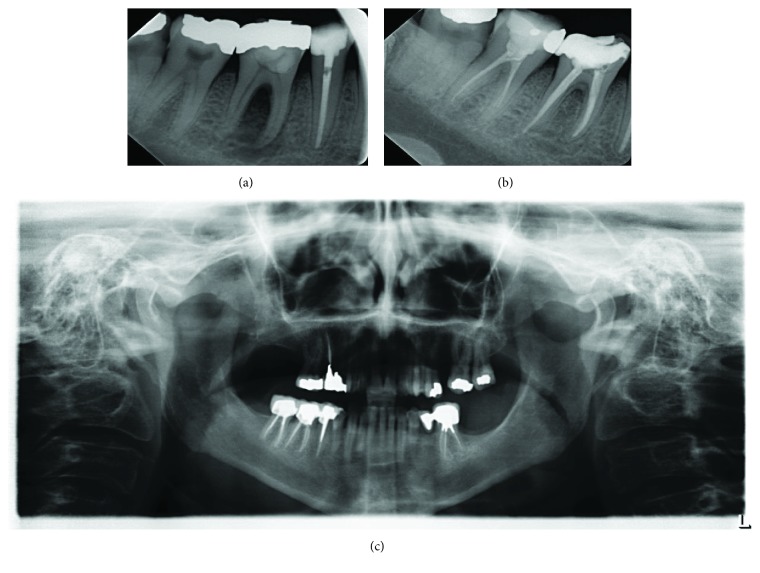
Case 1. (a) The initial radiograph of tooth #46 shows periapical and furcation bone resorption. (b) The 1-year recall radiograph shows healing of the bone lesion. (c) The 6-year follow-up radiograph shows tooth #46 with a permanent crown.

Endodontic treatment was accomplished in two visits with calcium hydroxide [Ca(OH)2] medication between appointments. Local anesthesia (1.8 mL of lidocaine with epinephrine 1 : 100,000) was administered, and the tooth was isolated with a rubber dam. An access opening was created and four canals were located. During the second visit, the localized swelling and sinus opening were thoroughly resolved, and the root canal treatment was completed with RaCe NiTi rotary files (FKG Dentaire, La Chaux-de-Fonds, Switzerland) and 5.25% sodium hypochlorite irrigation. The tooth was obturated with lateral condensation of gutta-percha and AH Plus sealer (AH Plus; Dentsply Maillefer, Tulsa, OK, USA). No periodontal treatment was administered. Follow-up X-ray images were obtained from 1 year to 6 years, which showed complete healing of the bone in the periapical and furcation areas (Figure 1).

### 2.2. Case 2: Iatrogenic Periodontal Lesion: Root Perforation

**Figure 2:**
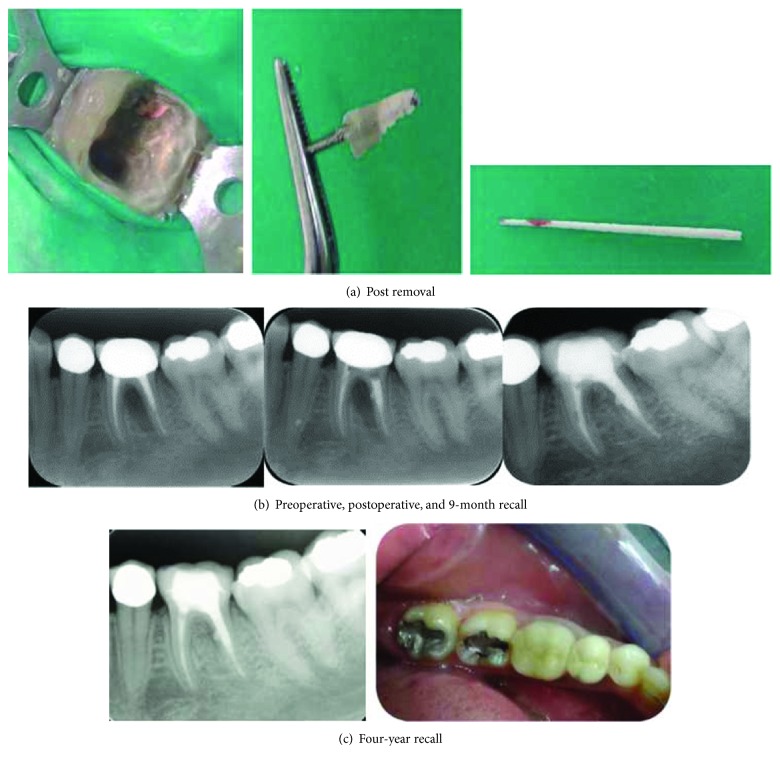
(a) Tooth #36 has a perforation in the distal root. The post is shown after removal, as is a paper point with a blood spot. (b) The mineral trioxide aggregate (MTA) repair. The recall examinations show osseous regeneration in the furcation. (c) The follow-up clinical photograph of tooth #36 shows the final crown and normal soft tissue.

A 30-year-old woman with a noncontributory medical history was referred to the endodontic specialist clinic at PSMMC complaining of pain and localized intraoral swelling in the left first mandibular molar. One year earlier, the tooth had undergone root canal treatment with cementation of the post and core and placement of a permanent crown. The clinical examination revealed localized swelling in the gingival sulcus. Periodontal probing through the furcation showed increased probing values with a grade II defect [2] (Figure 2). The radiographic examination revealed a large post in the distal canal and a large furcal lesion related to the distal root opposite the post placement. Iatrogenic root perforation was suspected.

After the administration of local anesthesia (1.8 mL of lidocaine with 1 : 100,000 epinephrine), the crown was removed with a crown removal instrument, and the post was removed with an ultrasonic instrument using a light brush and cutting motion to break up the cement around the post. A paper point was used to check for the presence of blood spots to determine the location and size of the perforation. The perforation site was irrigated with 2.5% sodium hypochlorite followed by drying of the canal and sealing of the perforation with mineral trioxide aggregate (MTA; Dentsply Tulsa Dental) mixed with saline and placed with a microapical placement system (Dentsply Tulsa Dental) and condensed with a paper point (Figure 2). A wet cotton pellet was then placed on the MTA material. The tooth was temporized with glass-ionomer cement and cementation of the crown. At the second visit, the cotton pellet was removed and the MTA setting was checked.

The patient was referred to the prosthodontics department for permanent crown placement. Follow-up appointments at 3 months, 6 months, 9 months, and up to 4 years showed complete healing of the soft tissue and bone lesions and a normal pocket depth of 3 mm.

### 2.3. Case 3: Iatrogenic Periodontal Lesions: Dental Injury/Trauma

**Figure 3:**
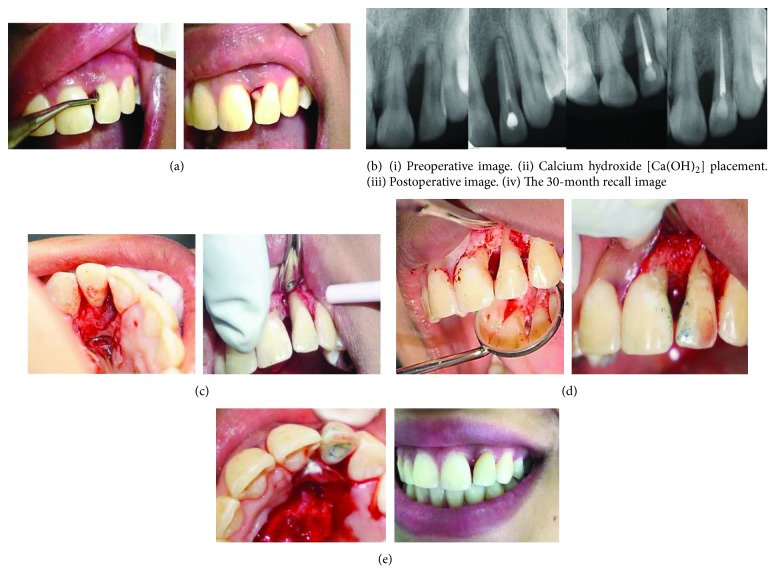
(a) A deep periodontal pocket and drainage of pus through the gingival sulcus are visible. (b) Radiographs of tooth #22 exhibit (i) a large lateral radiolucency on the mesial tooth surface extending from the bone crest to the root apex, (ii) calcium hydroxide [Ca(OH)2] placement, (iii) root canal obturation, and (iv) bone healing. (c) Surgical exposure of tooth #22 shows calculus accumulation and granulation tissue mesially with the absence of buccal bone. (d) Removal of calculus and granulation tissues and placement of the bone graft. (e) Placement of the barrier membrane; the 30-month recall photograph shows normal gingival tissue.

A 27-year-old woman with a noncontributory medical history was referred from the orthodontic department at PSMMC complaining of pus discharge from the gingival sulcus and slight gingival swelling on the palatal side opposite tooth #22 (i.e., maxillary left lateral incisor) after orthodontic treatment. Clinical and radiographic examinations revealed a sound tooth #22 with a mobility of grade II and a deep periodontal pocket > 10 mm mesial to tooth #22 with pus discharge from the pocket (Figure 3(a)). The thermal vitality test demonstrated a negative response. Moreover, the area was tender to percussion and palpation. Methylene blue stain was applied to exclude the presence of a crack or root fracture. It showed a negative result. The radiographic examination showed advanced bone resorption extending from the mesial bone crest toward the apex of tooth #22 (Figure 3(b)).

The diagnosis was necrotic pulp due to trauma during orthodontic treatment with symptomatic apical periodontitis. The first stage of the treatment plan was endodontic treatment, which was performed during two visits with calcium hydroxide medication between appointments (Figure 3(b)). Chemicomechanical debridement was administered with the ProFile.04 and ProFile.06 Taper Series 29 rotary instruments (Tulsa Dental Products) and sodium hypochlorite. The canal was obturated with lateral condensation of gutta-percha and AH Plus sealer (Dentsply Maillefer, Tulsa, OK, USA) (Figure 3(b)). The second stage of the treatment plan, which was a surgical procedure, was performed after the permanent composite restoration and a follow-up period of 3 months. Before surgery, the patient signed a consent form.

Local anesthesia (two 1.8 mL carpules of 2% lidocaine with 1 : 100,000 epinephrine) was then administered labially and palatally. A mucoperiosteal flap was raised mesially to the upper left canine tooth, and two vertical releasing incisions were formed in the anterior palatal area opposite teeth #21 and #23. A horizontal incision was formed from the left maxillary central incisor to the left maxillary canine. After flap reflection, a sling suture was placed in the tissue flap to secure it with the premolar tooth on the opposite side of the maxillary arch to aid the surgeon in improving visual and operative access by eliminating the need to manually retract the flap in the palatal area (Figure 3(c)) [8]. Cortical bone was absent on the mesial side of tooth #22. The root surface was covered with black calculus and the area was obliterated with granulation tissues. After removing the granulation tissues and calculus from the root surface with ultrasonic tips (Figures 3(c) and 3(d)), bone graft material (Puros Particulate Allograft; Zimmer Biomet Dental) mixed with saline was placed into the bony defect with a plastic instrument, and a resorbable barrier membrane (CopiOs membrane, Zimmer Biomet Dental) was placed above the bone graft. The flap was then repositioned and sutured with 4-0 Vicryl thread (Ethicon Inc., Somerville, NJ, USA). The patient was prescribed Augmentin (amoxicillin (875 mg) and clavulanic acid (125 mg)) (GlaxoSmithKline (Ireland) Ltd.) 1 g twice daily for 5 days and ibuprofen (600 mg) orally every 6 h for 2 days. Follow-up of the patient showed no tooth mobility, a reduced pocket depth of 4 mm, and healing of the soft and hard tissues (Figure 3(e)).

## 3. Discussion

The management of endodontic-periodontal lesions is a true challenge for the dentist because of the deleterious effects on the tooth structure and the supporting periapical structures (i.e., bone and periodontal membrane). The key to success in treating these cases depends on taking a correct history to determine the cause and reach an exact diagnosis of the case development. In addition, a clinician's ability to classify a lesion makes the treatment strategy or protocol very clear and precise. In this report, several successful endodontic-periodontal lesion cases were presented with different treatment modalities and classified according to the new classification system reported by Al-Fouzan in 2014 [4]. The classification of the first case was a primary endodontic lesion with drainage through the periodontal ligament through a fistula. The pulp was necrotic and there was a deep and narrow pocket in one tooth aspect. All of these factors confirmed the diagnosis and led to the correct treatment modality, which was endodontic treatment alone. The other cases were diagnosed as iatrogenic periodontal lesions that resulted from the treatment modality: by tooth perforation (as shown in the second case) or by dental trauma caused by orthodontic treatment, which is not mentioned in the Al-Fouzan classification [4]. The authors of the current study suggest adding this category to Al-Fouzan's classification under “iatrogenic periodontal lesions” because trauma from orthodontic movement can cause pulp necrosis and thereby lead to periodontal disease and pocket formation.

Root perforation is an unnatural communication between the root canal system and the supporting tissues of the teeth or the oral cavity [9]. One study [10] reported that 53% of iatrogenic perforations occur during the insertion of posts. Factors that affect the perforation prognosis depend on the location of the perforation, its duration, size of the perforation and tooth, sterilization of the perforation site, and the material used to seal the perforation. Immediate sealing of small perforations away from the coronal attachment of the tooth under aseptic conditions with compatible materials are the most important factors for a good prognosis [11, 12].

Mineral trioxide aggregate is a bioceramic material, composed of tricalcium silicate, tricalcium aluminate, tricalcium oxide, and silicate oxide, that forms a colloidal gel on hydration and solidifies in approximately 3 hours. The calcium oxide in MTA reacts with tissue fluids to form calcium hydroxide, which may then encourage hard tissue deposition because of its high pH. In addition, several studies [13–15] have demonstrated the sealing ability of MTA.

Dental pulp is a soft connective tissue encased in a rigid, noncompliant chamber; therefore, changes in pulpal blood flow or vascular tissue pressure can have serious effects on the health of the pulp. Many studies [16, 17] have explained the effects of orthodontic forces on teeth. These forces influence blood flow and cellular metabolism and lead to degenerative and/or inflammatory responses in the dental pulp. Spector et al. [18] reported two cases in which teeth were devitalized during orthodontic therapy. Hamersky et al. [19] used a radiorespirometric method to demonstrate a significant depression in the pulpal respiratory rate when a tooth underwent orthodontic movement. In addition, as a person's age increased, the relative amount of pulpal respiratory rate depression increased. Moreover, orthodontic forces may induce more rapid aging processes within the pulp because of blood flow interruption, and thereby reduce the pulp's ability to withstand future forces [16]. Orthodontically treated teeth also show histologic findings similar to those of periodontally involved teeth.

In endodontic-periodontal lesions, the cause of the lesion is endodontic or periodontal in origin, and the patient may benefit from undergoing root canal therapy first [20, 21] because pulpal infection could promote marginal epithelial downgrowth along the root surfaces of the teeth [22]. Unhealed lesions with persistent infection were further managed through endodontic-periodontal surgery or through periodontal regenerative surgery alone. This sequence of treatment conveys a good chance for primary healing, a better assessment of the periodontal conditions of the involved teeth, and controls reinfection by bacteria or their by-products [20].

Regenerative techniques involve cell differentiation, cell proliferation and induction, and/or tissue formation conduction. All of these factors work together to complete the healing of damaged periapical tissues. This healing can be obtained by using bone grafts (e.g., autografts, allografts, alloplasts, and xenografts), periodontal membranes (e.g., nonabsorbable or absorbable of the natural or synthetic type), growth factors, or a combination of these [7, 23].

In the first two cases, soft and hard tissue healing commenced with administering root canal therapy alone or perforation repair. However, in the third case, follow-up for 3 months after completing the root canal revealed a persistent bone defect and periodontal pocket, which indicated the need for a further treatment modality to restore the health of the periodontal tissues.

Von Arx and Cochran [24], Dietrich et al. [25], and Kim and Kratchman [26] described various classifications of periradicular lesions to ensure the best treatment modalities for such lesions. The last classification was reported by Von Arx and AlSaeed in 2011 [27] and is based on the type of periradicular lesion. Their classification was as follows: (i) the lesion is limited to the periapical area; (ii) the lesion erodes the lingual/palatal cortex (with or without erosion of the buccal cortex) and has caused a through-and-through (i.e., tunnel) defect; and (iii) the apicomarginal lesion has complete denudation of the buccal root surface. Investigators in clinical and experimental studies have concluded that only tunnel and apicomarginal defects could benefit from a regenerative treatment protocol [28, 29]. In the third case, there was an absence of buccal bone at tooth #22 after surgical exposure. Thus, the labial root surface was exposed to the root apex. The root surface was covered with calculus and granulation tissues. The calculus and granulation tissues were removed with ultrasonic scaling, followed by placing a bone graft and barrier membrane, which led to complete healing. Some studies have reported considerable success rates in managing such apicomarginal defects [26, 30, 31].

Controversial findings challenge the current understanding of the ideal interval between endodontic treatment and periodontal surgery. It has been reported that root canal treatment performed 10 weeks, 3 months, or 6 months before periodontal surgery did not impair periodontal healing [32–34]. In the third case, root canal treatment was performed 3-4 months before the periodontal surgery and showed no disruptive effect on complete bone healing. In conclusion, all cases presented in this report were successfully treated and showed great promise in managing endodontic-periodontal lesions, which are a very challenging disease condition that requires all possible treatment modalities reported in the literature such as endodontic therapy, periodontal therapy, and regenerative procedures to ensure satisfactory and complete healing. In this paper, we have added a modification to the latest endodontic-periodontal classification and highlighted the importance of using a step-by-step systematic approach for managing such complex lesions.
